# HoSAGE: sarcopenia in older patient with intermediate / high-risk prostate cancer, prevalence and incidence after androgen deprivation therapy: study protocol for a cohort trial

**DOI:** 10.1186/s12885-021-09105-8

**Published:** 2022-01-18

**Authors:** Anne-Laure Couderc, Patrick Villani, Julie Berbis, Emilie Nouguerède, Dominique Rey, Dominique Rossi, Éric Lechevallier, Delphine Badinand, Cyrille Bastide, Gilles Karsenty, Romain Boissier, Kahena Amichi, Xavier Muracciole

**Affiliations:** 1 Internal Medicine, Geriatrics and Therapeutic Unit, Sainte-Marguerite Hospital, AP-HM, 270 Boulevard Sainte Marguerite, 13009 Marseille, France; 2 Unit of Coordination in Onco-Geriatry (UCOG), PACA-west, Marseille, France; 3 CNRS, EFS, ADES, Aix-Marseille University, 264 Rue Saint Pierre, 13385 Marseille, cedex 05 France; 4 Epidemiology and Health Economics Unit, La Timone Hospital, AP-HM, 13385 Marseille, cedex 05 France; 5 Urological Surgery Unit, Nord Hospital, AP-HM, Chemin des Bourrely, 13915 Marseille, cedex 20 France; 6 Urological Surgery and Renal Transplantation Unit, Conception Hospital, AP-HM, 147 Boulevard Baille, 13005 Marseille, France; 7 Aix Marseille University, Marseille, France; 8 Radiotherapy Unit, Timone Hospital, AP-HM, 264 Rue Saint Pierre, 13385 Marseille, cedex 05 France; 9 Direction de la Recherche Clinique et de l’Innovation (DRCI), Assistance Publique des Hôpitaux de Marseille (AP-HM), 80 Rue Brochier, 13354 Marseille, Cedex 05 France

**Keywords:** Sarcopenia, older patient, prostate cancer, prevalence, incidence, androgen deprivation therapy, radiotherapy

## Abstract

**Background:**

Sarcopenia is defined by a loss of muscle strength associated to a decrease in skeletal muscle mass. Ageing greatly contributes to sarcopenia as may many other factors such as cancer or androgen deprivation therapies (ADT). This cohort study aims to evaluate (1) the prevalence of muscle disorders and sarcopenia in older patients before initiation of intermediate to high risk prostate cancer treatment with ADT and radiotherapy, and (2) the occurrence and/or aggravation of muscle disorders and sarcopenia at the end of cancer treatment.

**Methods:**

This cohort study is monocentric and prospective. The primary objectives are to determine the risk factor of sarcopenia prevalence and to study the relationship between ADT and sarcopenia incidence, in patients 70 years and older with histologically proven localized or locally advanced prostate cancer, addressed to a geriatrician (G8 score ≤14) for comprehensive geriatric assessment (CGA) in Marseille University Hospital. Secondary objectives encompass, measurement of sarcopenia clinical criteria along prostate oncological treatment; evaluation of the quality of life of patients with sarcopenia; evaluation of the impact of socio-behavioral and anthropological factors on sarcopenia evolution and incidence; finally the evaluation of the impact of ADT exposure on sarcopenia. Sarcopenia prevalence was estimated to be between 20 and 30%, therefore the study will enroll 200 patients.

**Discussion:**

The current guidelines for older patients with prostate cancer recommend a pelvic radiotherapy treatment associated to variable duration (6 to 36 months) of ADT. However ADT impacts muscle mass and could exacerbate the risks of sarcopenia. Our study intends to assess the specific effect of ADT on sarcopenia incidence and/or worsening as well as to estimate sarcopenia prevalence in this population. The results of this cohort trial will lead to a better understanding of sarcopenia prevalence and incidence necessary to further elaborate a prevention plan.

**Trial registration:**

The protocol was registered to the French drug and device regulation agency under the number 2019-A02319-48, before beginning the study (11/12/2019). The ClinicalTrials.gov identifier is NCT04484246, registration on the ClinicalTrials.gov (https://clinicaltrials.gov/ct2/show/NCT04484246).

## Background

Prostate cancer is the most common cancer for men over 60 years old worldwide according to 2020 Globocan’s data [1, 2]. For intermediate or high risks forms of prostate cancer, radiotherapy associated to androgen deprivation therapy (ADT) for 6 to 36 months is the reference treatment [3–5]. ADT induces several side effects including, anemia, alteration of physical functions, decrease of quality of life and activity [6]; in parallel, androgen inhibitors are known to be associated with metabolic effects that could result in obesity, diabetes, cardiovascular afflictions, sarcopenia or osteoporosis [7–9].

Sarcopenia is a degenerative muscle disorder characterized by loss of muscle strength and skeletal muscle mass, with or without an increase of fat mass (sarcopenic obesity) [9]. Aging, but also poor nutrition, inactivity or cancer can contribute to sarcopenia. Moreover, sarcopenia is a risk factor of early death, falls and loss of independence or/and mobility in older populations [9].

Sarcopenia screening and diagnosis are based on a combination of measures of muscle mass and muscle strength that vary according to the international guidelines and consensus [10–12]. Measures of physical performance are used to appreciate the severity of the disease.

Sarcopenia incidence in older patients is poorly known but awareness is growing among clinicians, as the criteria to diagnose malnutrition for patients 70 years and older, edited by the French High Health Authority (HAS) [13], just recently were amended to include sarcopenia. The diagnosis is based on the new updated recommendations of the revised European guidelines (EWGSOP2) [10]. Sarcopenia screening in not yet part of the guidelines on older patients’ cancer management. In this particular context, our study aims to document (1) the prevalence of muscle disorders and sarcopenia in old patients before initiation of prostate cancer treatment with ADT and radiotherapy, and (2) the occurrence and/or aggravation of muscle disorders and sarcopenia at the end of cancer treatment.

## Methods / Design

### Design

This monocentric cohort study will assess the prevalence of sarcopenia in 200 men with localized or locally advanced prostate cancer treated by radiotherapy associated with androgen deprivation therapy. The protocol was designed in respect of the recommendations of the Standard Protocol Items: Recommendations for Interventional Trials (SPIRIT) statement.

### Setting

Patients will be screened in the Radiotherapy Department, the Urology Department, the Urologic Surgery and Transplantation Department and finally in the Internal Medicine, Geriatrics and Therapeutic Department of Marseille University Hospital (AP-HM). Epidemiology and Health Economics Unit, will take part in the methodology and will perform the data analysis.

The protocol version 2 of the 21^st^ October 2019 was accepted by the committee of persons’ protection “Ile de France IV” on the 24/10/2019, first patient was enrolled on the 11/12/ 2019.

### Participants

**Fig. 1:**
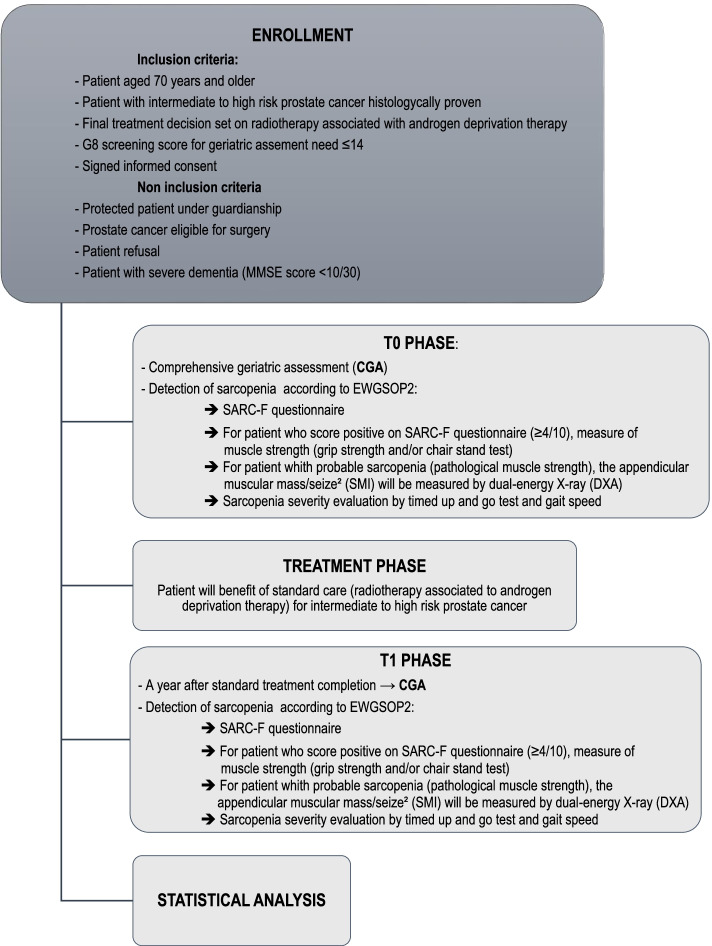
HoSAGE Study Flow Chart

All consecutive patients with intermediate or high risk prostate cancer addressed for CGA, who fit the inclusion/exclusion criteria and consent to participation to the trial, will be enrolled. The details of the inclusion and exclusion criteria are provided in Fig. 1. The main inclusion criteria are patients over 70 years old with histological diagnosis of localized or locally advanced prostate cancer according to D’Amico classification [14] with treatment decision set on radiotherapy associated to ADT; requiring a CGA based on the SIOG recommendations (G8 score ≤14 )[15].

The main exclusion criteria are patients under 70 years old, patients with metastatic prostate cancer showing a Mini-Mental State Examination (MMSE) score [16] under 10 out of 30.

### Recruitment and follow-up

#### Screening and enrollment - T0

Eligible patients will be identified by the radiotherapist and/or the urologist and/or the geriatrician. Patient over 70 years old screened with a G8 score ≤ 14/17 will be addressed to the geriatrician and benefit from full CGA and sarcopenia assessment: sarcopenia risk will be evaluated using the SARC-F questionnaire (Strength, Assistance walking, Rise from a chair, Climb stairs, and Falls questionnaire). Muscle strength will be assessed for patient at risk of sarcopenia (impaired SARC-F). For patient with probable sarcopenia (impaired muscle strength), sarcopenia diagnosis will be confirmed by measuring the skeletal muscle index (SMI). The severity of sarcopenia will also be evaluated.

#### Treatment Phase

Patient will benefit from standard cancer treatment for localized and locally advanced prostate cancer, i.e.: radiotherapy associated to ADT 6 to 36 months [4, 5]. Treatment phase will be handled by urologist and radiotherapist.

#### Follow-up – T1

A year after standard treatment completion, patient will once again benefit from CGA and sarcopenia assessment as described for T0.

Total duration of study will be 48 months, recruitment period will be 24 months and patient follow-up period, 12 months. A year after ADT end, patient will be contacted either by the geriatrician or the study coordinator to schedule the follow-up (T1). The study procedure and data collection are detailed in Table 1.

**Table 1:** HoSAGE Study schedule of activities and procedures

	T0	Treatment phase	T1
Oncologist consultation	X	X	
Diagnosis	X		
G8 score ≤14	X		
Geriatrician consultation	X		X
Enlighten consent	X		X
Enrollment	X		X
CGA	X		X
Demographic information	X		X
Social isolation	X		X
Cognitive status (MMSE and clock drawing test)	X		X
Mood status (GDS)	X		X
Autonomy (ADL and IADL Score)	X		X
Nutritional status (BMI, MNA score and Albumin)	X		X
Mobility (OLGT, Gait speed)	X		X
Sarcopenia screening (SARC-F, Handgrip test and chair stand test)	X		X
If Sarcopenia risk suspected (SARC-F≥4/10), and Muscle strength is impaired, SMI will be measured by (DXA)^a^	X^a^		X^a^
Radiotherapy treatment (dates, dose delivered and number of fractions)		X	
Androgen deprivation therapy (dates, dose delivered, frequency, adverse events)		X	

^a^DXA assessment performed only if SARC-F score is ≥4/10 and muscle strength is impaired (either handgrip strength under 27 kg for men and 16kg for women or five chair stand test is performed in more than 15 seconds)

*CGA: Comprehensive Geriatric Assessment; MMSE: Mini Mental State Examination; GDS: Geriatric Depression Scale; ADL: Activity of Daily Living; IADL: Instrumental Activity of Daily Living; BMI: Body Mass Index; MNA: Mini Nutritional Assessment; OLGT: One Leg Balance Test; SARC-F: Strength, Assistance walking, Rise from a chair, Climb stairs, and Falls; SMI: Skeletal Muscle Index (appendicular muscular mass/seize*^*2*^*); DXA: Dual-energy X-ray*

### Endpoints/Evaluation criteria

#### Primary endpoint

The primary endpoint is:

- The evaluation of sarcopenia prevalence for patients 70 years and older with localized or locally advanced prostate cancer before treatment initiation.

The geriatrician will screen for suspicion of sarcopenia using the SARC-F questionnaire and muscle strength assessment as recommended by the EWGSOP2 guidelines [10]. Patient whose SARC-F score is superior or equal to four out of ten are at risk of sarcopenia and will undergo muscle strength assessment. Patient with impaired muscle strength (either handgrip strength under 27 kg for men and 16kg for women or five chair stand in more than 15 seconds) are considered with probable sarcopenia [10]. To ascertain sarcopenia diagnosis, SMI will be estimated using the Appendicular Skeletal Muscle Mass (ASMM) measured with dual-energy X-ray absorptiometry (DXA) [17], adjusted for body size (ASMM/height^2^). Chosen cut-off point for low SMI indicating sarcopenia diagnosis as per EWGSOP2 guidelines is <7.0 kg/m2 [10]. Sarcopenia severity will be evaluated using Timed up and Go test and Gait speed assessment.

#### Secondary endpoints

The secondary endpoints are:

- The evaluation of sarcopenia incidence in patients 70 years and older with localized or locally advanced prostate cancer a year after the end of ADT. Sarcopenia assessment will be lead at enrollment and a year after ADT end.
- Evaluate the evolution of the physical performances by the measurement of mobility (Timed Up and Go test, gait speed and chair stand test) at enrollment and after ADT in patients 70 years and older with localized or locally advanced prostate.
- Evaluate quality of life (QoL) evolution before and after ADT for patient 70 years and older with localized or locally advanced prostate with or without sarcopenia. QoL will be evaluated using EORTC QLQ-c30 questionnaires and ELD-14 module at enrollment and a year after ADT end.
- Evaluate impact of socio-behavioral, anthropological and geriatric factors on sarcopenia prevalence for patients 70 years and older with localized or locally advanced prostate and on its incidence after ADT. Geriatric factors such as Autonomy (score <6 on Activity of Daily Living (ADL) Scale and score <4 on Instrumental Activity of Daily Living (IADL) scale) [18], nutrition (Weight, Body Mass index (BMI), Albumin, Mini Nutritional Assessment (MNA) )[19]., cognitive status (Mini Mental State Examination (MMSE)) [16], mood (Geriatric Depression Scale (GDS)) [20], polypharmacy, and comorbidities will be assessed by CGA. Demographic information such as age, gender, presence of a caregiver, urban or countryside way of living and physical activity (number of days when patient exercise an hour at least).
- Evaluate impact of ADT of patients 70 years and older with localized or locally advanced prostate on sarcopenia. Oncological data such as cancer stage and anatomy; testosterone and PSA levels at enrollment and a year after ADT end; treatment protocol for radiotherapy and ADT will also be collected.

### Statistical considerations

#### Sample size, power, and statistical methods

Under the assumption that the prevalence of sarcopenia in older patients with prostate cancer before initiation of treatment with ADT is between 20 to 30%, according to one of the rare studies published on the subject [21], a sample size of 200 subjects allows an estimation with a width of two-sided 95% confidence interval between 0,115 to 0.131. Previous feasibility analysis showed current collaboration between urologist, radiotherapist and geriatrician should allow enrollment of the adequate number of patients during the trial period.

#### Data analysis

The data will be analyzed using SPSS version 17.0 software. Method and analysis will be based on the Consolidated Standards Of Reporting Trials Statement (CONSORT) (http://www.consort-statement.org/consort-statement/). The patients who would be inappropriately included despite providing consent and patients who removed their consent to participate won’t be included in the final analysis. The full population (including all subjects who will be enrolled and will be at least evaluated at T0) will be included in the analysis of primary endpoint. The normality of the parameters will be estimated using frequency histograms and the Shapiro test; the baseline parameters will be presented according to the timeline of assessments: ‘Enrollment – T0’ and ‘A year after ADT end – T1’.

The SMI under 7 kg/m^2^ at enrollment and /or a year after ADT end is considered as a proven sarcopenia diagnosis. Along with incidence of sarcopenia, we will monitor evolution of SMI and/or of physical performances (handgrip strength and/or chair stand test) for diagnosed sarcopenic patient. Only patients with probable sarcopenia (SARC-F score ≥4/10 and then muscle strength impairment), will benefit from a DXA exam [10, 22, 23]. To evaluate incidence, enrollment (T0) will be considered as baseline and compared to T1 data. Changes between initial SMI and/or handgrip strength and/or chair stand test will be compared between the two groups, and the analysis of variance for repeated measurements will be performed to compare the changes in the scores over time between the 2 groups. Multivariate analysis using linear regression models will be performed to determine the association between our analyzed variables and sarcopenia occurrence. Variables relevant to the models will be selected based on their clinical significance (two-tailed with 5% significance level). The secondary endpoints will be compared for all the patients between the 2 assessments (Chi2 test or Fisher’s exact test for categorical variables and Student’s t test for continuous variables).

## Discussion

Sarcopenia is an independent risk factor for adverse events as falls, increased hospital length of stay or readmission and death [24]. Prevalence of sarcopenia in the community was 1-33% with higher prevalence in older or acutely ill patients treated in care facilities. European Working Group on Sarcopenia in Older People (EWGSOP), discriminates two types of sarcopenia: primary, when non other cause than old age can be identified; secondary to physical inactivity (bed rest, immobility, sedentary lifestyle), to disease (like organ deficiency, inflammatory diseases, endocrinopathies or cancer) or to nutrition disorders (low protein or energy intakes, micronutrient deficiency …) [9].

Older patients are particularly at risk of sarcopenia as muscle mass decrease with age [11] and that protein-energy malnutrition is common in this age group [25].

Likewise patients with cancer often suffer malnutrition as a result of metabolic changes, mechanical blockages or abnormalities, side effects of treatment or psychosocial issues [26]. Even though malnutrition is less present in prostate cancer [27], androgen deprivation treatment leads to hypogonadism causing undesirable changes in lipid, glucose, muscle or bone metabolism leading to accelerated bone loss, osteoporosis and increased fracture risks [7, 28]. In a preliminary study we showed that muscle disorders were present before initiation of ADT in older patients with prostate cancer and an increased risk of falls at the end of ADT [29].

The aim of the HoSAGE study protocol is to evaluate the actual prevalence of sarcopenia in intermediate to high risk prostate cancer patients over 70 years old, and the incidence on sarcopenia in this population following ADT associated to radiotherapy. Using the most recent screening tool to detect sarcopenia (SARC-F) [10], the results of this study may lead to increment ADT with sarcopenia preventions programs.

## Trial status

At the time of manuscript submission, the status of the trial is ‘recruiting’

## Data Availability

Not applicable

## List of abbreviations

ADT: Androgen Deprivation Therapy
G8 ONCODAGE: scale for detection of geriatric frailties in cancer patients over 70 years old
CGA: Comprehensive Geriatric Assessment
EWGSOP: European Working Group on Sarcopenia in Older People
AP-HM: Assistance Publique-Hôpitaux de Marseille
ADES: Anthropology Bio-cultural, Law, Ethic and Health laboratory
CNRS: National Center of Scientific Research
SIOG: International Society of Geriatric Oncology
MMSE: Mini-Mental State Examination
SARC-F: Strength, Assistance walking, Rise from a chair, Climb stairs, and Falls questionnaire
DXA: dual-energy X-ray absorptiometry
SMI: skeletal muscle index
ADL: Activity of Daily Living
IADL: Instrumental Activity of Daily Living
BMI: Body Mass Index
MNA: Mini Nutritional Assessment
GDS: Geriatric Depression Scale
QoL: Quality of life
EORTC QLQ-c30: European Organization for Research and Treatment of Cancer Quality of Life form c30
EORTC QLQ-14 ELD: European Organization for Research and Treatment of Cancer complementary module of Quality of Life form c30, ELD-14 dedicated to elderly cancer patients
CONSORT: CONsolidated Standards Of Reporting Trials
